# Turning Down the Thermostat: Modulating the Endocannabinoid System in Ocular Inflammation and Pain

**DOI:** 10.3389/fphar.2016.00304

**Published:** 2016-09-15

**Authors:** James T. Toguri, Meggie Caldwell, Melanie E. M. Kelly

**Affiliations:** ^1^Department of Pharmacology, Dalhousie University, HalifaxNS, Canada; ^2^Department of Ophthalmology and Visual Sciences, Dalhousie University, HalifaxNS, Canada; ^3^Anesthesia, Pain Management & Perioperative Medicine, Dalhousie University, HalifaxNS, Canada

**Keywords:** cannabinoids, eye, inflammation, pain, ocular inflammation, ocular pain, corneal inflammation, ocular cannabinoids

## Abstract

The endocannabinoid system (ECS) has emerged as an important regulator of both physiological and pathological processes. Notably, this endogenous system plays a key role in the modulation of pain and inflammation in a number of tissues. The components of the ECS, including endocannabinoids, their cognate enzymes and cannabinoid receptors, are localized in the eye, and evidence indicates that ECS modulation plays a role in ocular disease states. Of these diseases, ocular inflammation presents a significant medical problem, given that current clinical treatments can be ineffective or are associated with intolerable side-effects. Furthermore, a prominent comorbidity of ocular inflammation is pain, including neuropathic pain, for which therapeutic options remain limited. Recent evidence supports the use of drugs targeting the ECS for the treatment of ocular inflammation and pain in animal models; however, the potential for therapeutic use of cannabinoid drugs in the eye has not been thoroughly investigated at this time. This review will highlight evidence from experimental studies identifying components of the ocular ECS and discuss the functional role of the ECS during different ocular inflammatory disease states, including uveitis and corneal keratitis. Candidate ECS targeted therapies will be discussed, drawing on experimental results obtained from both ocular and non-ocular tissue(s), together with their potential application for the treatment of ocular inflammation and pain.

## Introduction

The ocular effects of cannabinoids have been studied extensively in animals and humans over the last few decades. These compounds generate a number of actions in the eye including: ocular hypotension and hyperemia, as well as modulation of visual function (reviewed in Yazulla, 2008; Cairns et al., 2016a; Kokona et al., 2016). It was not until the 1990’s, however, that the effects of cannabinoids in the eye were formally ascribed to actions on the ocular ECS (reviewed in Yazulla, 2008). Components of this system, including endocannabinoid ligands that act at two cloned cannabinoid receptors, CB_1_R and CB_2_R, and cognate enzymes involved in endocannabinoid biosynthesis and degradation, are present throughout ocular tissues in all species studied to date, including humans and non-human primates (Porcella et al., 1998; Straiker A. et al., 1999; Straiker A.J. et al., 1999; Porcella et al., 2000; Hu et al., 2010; Bouskila et al., 2012, 2013; Cécyre et al., 2013, 2014).

The ocular hypotensive effects of cannabinoids, specifically, have generated considerable interest over the last few decades largely due to their potential use in the treatment of glaucoma. Glaucoma is a blinding eye disease characterized by a progressive painless loss of vision, for which IOP is a primary risk factor (reviewed in Tomida, 2004; Nucci et al., 2007; Pinar-Sueiro et al., 2011; Cairns et al., 2016a). The IOP lowering actions of cannabinoids arise primarily via interactions at CB_1_R that are localized to anterior eye tissues involved in the production and outflow of aqueous humor (Straiker A. et al., 1999; Straiker A.J. et al., 1999; Hudson et al., 2011). Additional retinal neuroprotective and decreased neuroinflammatory responses have also been reported in the eye for cannabinoids acting at CB_1_Rs (Pryce et al., 2003; Rossi et al., 2011; reviewed by Cairns et al., 2016b).

Despite evidence for reducing IOP and potential neuroprotective benefits, the medical community has not embraced the use of cannabinoids as a clinical treatment for glaucoma (Järvinen et al., 2002; Buys and Rafuse, 2010; Novack, 2016). This was documented by the Canadian Ophthalmological Society in a full policy statement released in 2010 (Buys and Rafuse, 2010) and by the American Academy of Ophthalmology in Schwab et al. (2014). This is largely because the actions of cannabinoids that act at CB_1_R in humans and experimental vertebrate animals produce transient alterations in IOP, are subject to tachyphylaxis, and can have both peripheral and CNS side-effects (Corchero et al., 1999; Chiou et al., 2013).

Both CB_1_R and CB_2_R mRNA and protein have been reported in the eye (reviewed in Yazulla, 2008; Bouchard et al., 2016; Cairns et al., 2016b). CB_1_R is expressed throughout the retina and is also is found in some anterior ocular tissues, including the trabecular meshwork (Straiker A.J. et al., 1999; Porcella et al., 2000; Stamer et al., 2001). In contrast to broader ocular distribution of CB_1_R, expression of CB_2_R in the eye is more limited and largely localized to resident immune cells and retinal glia in primates (Bouskila et al., 2013), although the CB_2_R has been reported in murine retina including the retinal pigment epithelium, photoreceptors, horizontal and amacrine cells and within the ganglion cell layer (López et al., 2011). CB_2_Rs have been implicated in aqueous humor turnover (Zhong et al., 2005). However, unlike CB_1_Rs, the IOP lowering actions of cannabinoids are still present in CB_2_R null animals (Hudson et al., 2010; Caldwell et al., 2013), arguing against a role for the use of CB_2_R agonists to regulate IOP. More recently, several publications have indicated that cannabinoids targeting CB_2_R may be therapeutically relevant in ocular inflammatory disease (Xu et al., 2007; Toguri et al., 2014, 2015). Additionally, several non-cannabinoid receptors, including GPR18 which binds the endogenous ligand *N*-arachidonoyl glycine, have also been implicated in the ocular actions of some cannabinoids (Caldwell et al., 2013).

The following review addresses the ocular immune response and presents recent evidence on ECS modulation of ocular inflammation. A specific focus of this review is on cannabinoid receptors as emerging non-steroidal targets for ocular inflammatory disorders, including uveitis and corneal keratitis and neuropathic pain, the latter of which lacks satisfactory treatment options. We present recent evidence that supports a prominent role for the CB_2_R as novel anti-inflammatory target in the eye. Additionally, the potential of targeting CB_1_Rs in corneal neuropathic pain is discussed, together with considerations for future research and possible limitations in the use of cannabinoids to treat ocular disease.

## The Ocular Immune System

**FIGURE 1:**
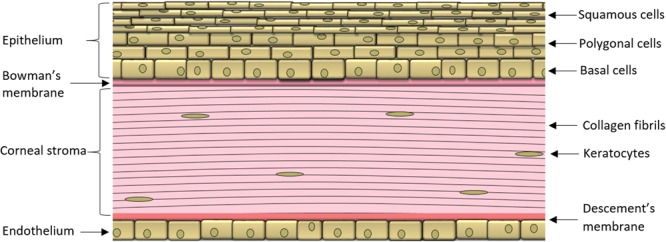
**The cornea. Schematic representation of the corneal layers in a cross section**.

During ocular inflammation, a deregulated immune response is detrimental to ocular tissue function and can have sight-threatening consequences (Suttorp-Schulten and Rothova, 1996; Dunn, 2015). The eye is a unique organ as it contains both the peripheral and CNS components of the immune system, although the two immune systems function by analogous mechanisms. Similar to most other organ systems in the body, the eye is privy to tissue resident macrophages and DCs that induce innate immune responses. These cell populations are localized to the cornea, iris, ciliary body, and choroid, while no immune cells are present in the aqueous humor and lens under normal physiologic conditions (McMenamin et al., 1994; Pouvreau et al., 1998; Li et al., 2013). The cornea is an avascular tissue comprised of an epithelial layer, stroma made of keratocytes and collagen interlaced with a dense network of nerves, followed by the endothelium (**Figure 1**; Marfurt et al., 2010; Shaheen et al., 2014). Specialized DCs called Langerhans cells are located in the corneal stroma (Akpek and Gottsch, 2003; Hamrah et al., 2003), while in the iris, both macrophages and major histocompatibility complex II class DCs are found in close proximity to the pupil and near the ciliary body (McMenamin, 1997). The choroid is highly populated with antigen presenting cells found throughout the stromal connective tissue (McMenamin, 1997). Mast cells have been localized in the choroid, and may play a role in the susceptibility to the development of inflammation in some experimental models (McMenamin, 1997).

This immune cell population is mirrored in the retina by microglia, macrophages while DCs are found within the connective tissue of the sclera (Eter et al., 2008; Kezic and McMenamin, 2008; London et al., 2013). The tissue resident populations of immune cells are outlined in **Table 1**. These cellular populations in the periphery are primarily divided between tissue resident macrophages and DCs which have the ability to phagocytize foreign bodies, release inflammatory mediators, recruit and present antigens to immune cells, and induce differentiation and proliferation of other cells. Although the eye has a collection of resident immune cells acting as sentinels and activators of the immune system, it also has a special condition known as immune privilege.

**Table 1:** Presence and location of ocular immune cells during non-pathological conditions.

	Cornea	Iris	Ciliary body	Trabecular meshwork	Choroid	Retina	Conjunctiva
Macrophages	X^M,R,H,G^ (Rodrigues et al., 1981) (Brissette-Storkus et al., 2002) (Hamrah et al., 2003)	X^M,R,^ (McMenamin et al., 1994; McMenamin, 1997)	X^M,R,H^ (McMenamin et al., 1994; McMenamin, 1997)		X^M,R,H^ (McMenamin et al., 1994; McMenamin, 1997)		X^H^ (Hingorani et al., 1997)
Dendritic cells	X^M^ (Hamrah et al., 2002)^H^(Vantrappen et al., 1985) (Hamrah et al., 2003)	X^M,R^ (McMenamin, 1997)	X^M,R^ (McMenamin, 1997)		X^M,R^ (McMenamin, 1997)		X^H^ (Hingorani et al., 1997)
Mast cells		X^+D,Ca,Ma,H^ (McMenamin, 1997)	X^H^ (McMenamin, 1997)		X^R,H^ (McMenamin, 1997)	X ^H^	
CD4+ T cells	X^H 1∗11^						X^H^ (Hingorani et al., 1997)
CD8+ T cells	X^H∗^(Vantrappen et al., 1985)						X^H^ (Hingorani et al., 1997)
CD3+ T cell	X^H ∗^(Vantrappen et al., 1985)						X^H^ (Hingorani et al., 1997)
Monocytes				X^H,Mo^ (Alvarado et al., 2010)			
Microglia						X^M, R,C^ (McMenamin, 1997; Langmann, 2007) (Karlstetter et al., 2010)

*^M^mouse, ^R^rat, ^H^human, ^G^guinea pig, ^C^chick, ^Ca^cat, ^Mo^monkey, ^Ma^marsupial, ^D^dog.*

## The Immune Privilege of the Eye: A Complicating Factor

Immune privilege of the eye is a group of processes that result in the suppression of inflammation to prevent tissue damage and loss of sight. This is made up of intrinsic factors including physical boundaries like the blood-aqueous and blood retinal barriers; in addition to active factors such as immunomodulatory proteins (Streilein et al., 2002). Active factors which contribute to the immune privilege, include the endogenous release of immunomodulatory factors into the aqueous humor such as transforming growth factor-β (TGF-β), immunoregulation via cell-to-cell contact mechanisms with corneal endothelium and iris pigmented epithelium, and antigen presenting cell development of antigen tolerance (Caspi, 2010; Taylor and Kaplan, 2010; London et al., 2013; Mochizuki et al., 2013). Together these adaptations help to maintain the ocular microenvironment and proper ocular function.

## Targeting the ECS to Treat Ocular Inflammatory Disease

Inflammatory eye diseases contribute to the global incidence of blinding eye disease, presenting a significant risk of vision loss and blindness as well as a high medical and economic burden on populations (Yu et al., 2011; Dunn, 2015). Collectively, these conditions encompass both intraocular inflammation (e.g., uveitis, uveoretinitis, proliferative, and vitreoretinopathy), as well as ocular surface inflammation, including corneal inflammation and neuropathology (Akpek and Gottsch, 2003; Belmonte et al., 2015; Dunn, 2015; Galor et al., 2015; Rosenthal and Borsook, 2015). Both the innate and adaptive immune responses can result in detrimental, sight limiting complications, albeit through different cellular mechanisms. Although the subset of immune cells is fundamentally different during innate and adaptive immunity, both result in immune cell migration, release of inflammatory mediators and ensuing tissue damage (Streilein, 2003; Taylor, 2009; Caspi, 2010; Mochizuki, 2010). In some instances pain is a co-morbidity of ocular inflammation. For example in iritis, keratitis, and corneal trauma, pain can be manifested as acute changes in nociception alone, or more prolonged alterations can occur, leading to chronic and neuropathic pain (Belmonte et al., 2015; Galor et al., 2015; Rosenthal and Borsook, 2015; Goyal and Hamrah, 2016).

Recent evidence suggests that the ECS could be a therapeutic target in the treatment of ocular inflammation. The effects of cannabinoids have been shown to be beneficial in several animal models of intraocular inflammation (Xu et al., 2007; Altinsoy et al., 2011; Toguri et al., 2014, 2015). These studies have included models of EIU in rabbit (Altinsoy et al., 2011), rat (Toguri et al., 2014, 2015), and EAU in mouse (Xu et al., 2007). Uveitis is an overarching term which typically describes inflammation of any part of the uvea (iris, ciliary body, and choroid). Inflammation can be strictly localized to uveal tissue or be more extensive and involve ocular structures including: sclera, retina, vitreous, and optic nerve (Jabs et al., 2005; Read, 2006; Larson et al., 2011). Anterior uveitis, is additionally associated with pain and photophobia (Smith et al., 1998; Dunn, 2015). EIU is the most commonly used model of intraocular inflammation and changes reported in this model are consistent with uveitis seen in humans, including release of inflammatory mediators, activation and recruitment of immune cells (specifically T-cells in EAU), disruption of the blood aqueous/ blood retinal barrier, vasculitis and granulomas (Streilein et al., 2002; Caspi, 2006; Taylor, 2009). EAU, a widely used animal model of uveoretinitis (posterior uveitis) (Caspi, 2006), is a specific form of uveitis associated with chronic autoimmune disorders and affects the retina.

The first study describing the use of cannabinoids for the treatment of intraocular inflammation, was conducted by Xu et al. (2007) in an EAU model. The selective CB_2_R agonist, JWH 133, significantly decreased inflammatory cell infiltration in the retina, reducing cellular infiltrates and granulomas in a dose-dependent manner. The authors attributed the effects of JWH 133 to inhibition of leukocyte migration to multiple pro-inflammatory stimuli. CB_2_R activation also inhibited T cell proliferation and associated cytokine production. JWH 133 was able to suppress antigen presentation, as antigen presenting cells from animals treated with the CB_2_R agonist inhibited T cell proliferation following co-culture with T cells (Xu et al., 2007).

Compared to the EAU model, cannabinoid actions have been described more extensively in EIU. In the EIU model, cannabinoid administration has been reported to be both pro-inflammatory (Altinsoy et al., 2011) and anti-inflammatory (Toguri et al., 2014, 2015). In the former study, administration of the endocannabinoid, anandamide, in a rabbit model of EIU, significantly increased the clinical score of intraocular inflammation and increased leukocyte counts and protein in the aqueous humor (Altinsoy et al., 2011). Injection of AM251, a CB_1_R antagonist, resulted in a complete ablation of neutrophils within the aqueous humor, and a reduction in histopathology. However, although AM251was able to decrease some measures of ocular inflammation, protein was still found in the aqueous humor and the clinical ocular inflammation score was not altered in the presence of the CB_1_R antagonist. The reduction in neutrophils in the aqueous humor was attributed to a CB1R driven reduction in cytokines as reported in other studies (Mnich et al., 2010).

In further support of a CB_2_R-mediated anti-inflammatory action of cannabinoids in the eye, Toguri et al. (2014) demonstrated that topical application of the CB_2_R agonist, HU308, decreased ocular inflammation in a rat EIU model. Activation of the CB_2_R reduced leukocyte-endothelial adhesion within the iridal microcirculation following LPS administration. The decrease in leukocyte-endothelial adhesion was attributed to the inhibition of pro-inflammatory mediators including TNF-α, IL-1β, IL-6 as well as a reduction in the mRNA levels of the transcription factors NF-κB and AP-1. These authors compared the anti-inflammatory actions of the CB_2_R agonist, HU308, to that of the steroids, dexamethasone and prednisolone, and the non-steroidal anti-inflammatory drug, nepafenac, all used clinically in the treatment of uveitis. Among these anti-inflammatory agents, HU-308 was superior in attenuating leukocyte-endothelial adhesion and reducing pro-inflammatory cytokines (Toguri et al., 2014).

In further support of an anti-inflammatory action of cannabinoids in the eye, Toguri et al. (2015) also demonstrated that the CB_1_R/CB_2_R agonist, WIN55212-2, was able to reduce leukocyte-endothelial adhesion within the iridial microcirculation following systemic administration of LPS. In this study, leukocyte adhesion was measured in vessels with a diameter of both greater and less than 25 μm in order to investigate the possibility of alterations in hemodynamics contributing to the cannabinoid effects. When only CB_2_Rs were activated, in the presence of selective inhibition of CB_1_Rs, leukocyte adhesion was still decreased in the iridial microvasculature. However, when CB_2_Rs were blocked, allowing only activation of CB_1_Rs, leukocyte adhesion was attenuated only in smaller vessels (<25 um), a finding which was attributed to increases in shear forces due to alterations in vascular hemodynamics. In keeping with an improvement in iridial microcirculatory function observed with cannabinoid treatment, studies of ocular blood flow using Laser Doppler technology indicated that activation of either, or both, CB_1_R or CB_2_R, reduced the decrease in local blood flow observed when LPS was given alone (Toguri et al., 2015).

Taken together, the majority of these studies (Xu et al., 2007; Toguri et al., 2014, 2015) provide strong evidence that cannabinoids, and more specifically, CB_2_R agonists, are efficacious treatments for ocular inflammation, even out-performing traditional therapies such as steroid and non-steroidal anti-inflammatory drugs. As CB_2_R is upregulated during inflammation, this receptor has potential as an excellent drugable target for immunosuppression during ocular inflammatory diseases.

## The ECS and Ocular Surface Inflammation and Pain

**FIGURE 2:**
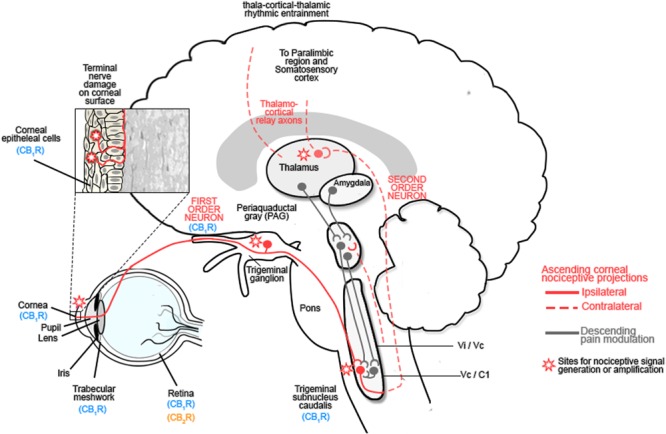
**The ocular sensory pathway.** The stroma of the cornea contains a dense network of nerve fibers expressing: mechanoreceptors, polymodal receptors and cold receptors. Following damage to the corneal epithelium, inflammatory mediators are released and immune cells are recruited to the site of injury. These inflammatory mediators include: cytokines, H^+^, ATP, adenosine, prostaglandins, leukotrienes, bradykinin, 5-HT, platelet-activating factor, histamine and neuropeptides, all of which contribute to the alteration in nociceptive signaling by activating Transient receptor protein (TRP) channels of nociceptors, which are responsible for nociceptor signaling (Rosenthal et al., 2009; Belmonte et al., 2015; Galor et al., 2015; Rosenthal and Borsook, 2015; Goyal and Hamrah, 2016). These first order neurons project to the trigeminal ganglion and synapse with second order neurons in the trigeminal subnucleus caudalis before projecting to the spinothalamic pathways and the thalamus or the periaqueductal gray (PAG). Third order neurons from the thalamus relay information to the somatosensory cortex and paralimbic region, while those from the PAG modulate trigeminal activity (Belmonte et al., 2015; Galor et al., 2015; Rosenthal and Borsook, 2015; Goyal and Hamrah, 2016). Activation of this pathway contributes to hyperalgesia and spontaneous pain by sensitizing corneal nociceptors. Chronic neuropathic pain results after the initial lesion has healed and occurs due to a decreased threshold of stimulation, spontaneous depolarization of nociceptors and altered excitability of voltage gated channels which modulate excitability of polymodal nociceptors. Reproduced and adapted with permission from Elsevier publishing group and Rosenthal et al. (2009). Original image from Rosenthal et al. (2009).

The corneal epithelium is densely innervated by sensory nerves that arise from the ophthalmic division of the trigeminal nerve (Marfurt et al., 2010; reviewed in Goyal and Hamrah, 2016). Stimulation of corneal nociceptors by noxious stimuli activates ion channels, such as TRPV1 channels, in sensory nerve endings to transduce sensory information to second order neurons in trigeminal brainstem complex before projecting via the thalamus to somatosensory cortex and paralimbic structures (**Figure 2**; reviewed in Belmonte et al., 2015; Goyal and Hamrah, 2016).

Damage to the cornea resulting from acute or chronic infectious and non-infectious disease, or trauma, results in the release of inflammatory mediators and immune cell recruitment, leading to improper tissue remodeling and peripheral sensitization. Injury-induced functional plasticity, with alterations in the threshold potentials for peripheral corneal nociceptors, amplifies peripheral pain signaling and can lead to central sensitization with increased pain perception (**Figure 2**; Rosenthal and Borsook, 2015; Goyal and Hamrah, 2016). Corneal inflammatory disease (keratitis) can result in corneal neuropathy (CNP) and is inadequately treated with current clinical agents (reviewed in: Rosenthal and Borsook, 2012; Belmonte et al., 2015; Galor et al., 2015; Rosenthal and Borsook, 2015; Goyal and Hamrah, 2016).

In support for a role for the ECS in the cornea, abundant CB_1_R protein is present in human and non-human primate cornea (Straiker A.J. et al., 1999) and recent evidence indicates mRNA for additional components of the ECS including the endocannabinoid, 2-AG, and enzymes which metabolize endocannabinoids such as, monoacylglycerol lipase, and alpha/beta-hydrolase domain 6 and 12, as well as, fatty-acid amide hydrolase and *N*-acylethanolamine-hydrolyzing acid amidase (Murataeva et al., 2015). Additionally, mRNA for G-protein coupled receptors GPR35, GPR55, and GPR92 was also detected in bovine cornea (Murataeva et al., 2015). These authors did not detect significant expression of CB_2_Rs under non-pathological conditions.

While the literature supports the efficacy of cannabinoids that act at both CB_1_Rs and CB_2_Rs in both acute and chronic pain, (Guindon and Hohmann, 2008; Yu et al., 2010; La Porta et al., 2013), the effects of cannabinoids on corneal pain specifically have not been extensively studied. One study, however, did investigate the effects of the non-selective CB_1_R/CB_2_R agonist, WIN55212-2 on activity in the rat trigeminal brainstem complex; corneal nerves terminate in two spatially distinct regions of the medulla, the trigeminal interpolaris/caudalis (Vi/Vc) transition and subnucleus caudalis/upper cervical cord (Vc/C1) junction region (Bereiter et al., 2002). These authors used topical mustard oil or a CO_2_ air puff to activate corneal nociceptors and found that WIN55212-2 reduced neural activity in the Vi/Vc transition but not in the Vc/C1 junction. The effects of WIN55212-2 were reduced by the CB_1_R antagonist, SR141716. Bereiter et al. (2002) concluded that CB_1_R activation affects the activity of corneal-responsive neurons that terminate in the Vi/Vc transition and preferentially contribute to homeostatic and reflexive corneal functions, e.g., blinking and lacrimation, rather than neurons that terminate in the Vc/C1 region and regulate sensory discrimination components of corneal nociception. Given that this study used only acute stimuli, further research examining the role of the ECS in chronic models of corneal neuropathic pain is needed to shed additional light on the functional role of the ECS in corneal pain.

Recently, several reports have emerged that indicate cannabinoid receptor modulation could have therapeutic efficacy for corneal epithelial cell proliferation and migration, effects which would be beneficial in promoting wound healing and treating corneal neuropathic pathologies, respectively (Yang et al., 2010, 2013; Murataeva et al., 2015). Yang et al. (2010), using HCEC, reported that stimulation of CB_1_R caused transactivation of EGFR and increases in transient intracellular [Ca^2+^]. EGFR is associated with mediating corneal epithelial renewal, via inducing cellular proliferation and migration. Furthermore, an increase in HCEC proliferation was observed when either CB_1_R or TRPV1 was activated.

In support of these findings, Yang et al. (2013) demonstrated co-localization of CB_1_R and TRPV1 in the mouse corneal epithelium. Using a corneal alkali burn model, these authors also reported that the cannabinoid agonist, WIN55212-2, improved wound healing and reduced stromal thickening, with full recovery of the epithelial layer at 5 days post injury. The immunosuppressive effects of WIN55212-2 in this study were attributed to desensitization of TRPV1 and occurred as a result of interactions between the CB_1_R and, TRPV1, that resulted in a WIN55212-2-mediated decrease in TRPV1-mediated phosphorylation of downstream signaling molecules transforming growth factor-β-activated kinase (TAK1) and c-Jun N-terminal kinase 1 (JNK1) activation, effects that were blocked by the CB_1_R antagonist, AM251. This report further indicated that administration of WIN55212-2 improved corneal ulceration, scarification and neovascularization compared to untreated animals and that wound closure was prolonged in CB_1_R^-/-^ mice.

A CB_1_R-mediated and ERK-dependent signaling mechanism for corneal epithelial cell migration was proposed by Murataeva et al. (2015) who reported that WIN55212-2-mediated chemotaxis of BCEC involved dephosphorylating of ERK1/2 and was blocked following treatment with the CB_1_R antagonist, SR141716. These authors also demonstrated that the endocannabinoid, 2-AG, induced chemotaxis of BCEC; however, this effect was only partially inhibited by SR141716, indicating that the chemotactic properties of 2-AG may arise via actions at a receptor distinct from CB_1_R. While this study provided evidence for CB_1_R-mediated corneal epithelial cell migration, unlike Yang et al. (2010), they failed to observe corneal epithelial cell proliferation and instead observed that the CB_1_R agonist, CP55940, antagonized the effects of EGF-mediated proliferation (Murataeva et al., 2015). Differences reported in this study compared to the study by Yang et al. (2013) were suggested to be the result of cell-specific variation or off-target effects due to the high concentration of cannabinoid drugs used. While clearly additional research is required to resolve the role for cannabinoid receptors in corneal epithelial cell growth and function, these studies highlight a potential role of CB_1_Rs in corneal wound healing.

## Conclusion

Evidence to date suggests a role for the ECS in mitigating ocular inflammation and pain. Although research in this area is still relatively limited, the potential for developing novel pharmacological tools exploiting the ECS for ocular inflammation and pain looks promising given that targeting both the CB_1_R and CB_2_R has proven beneficial in models of intraocular inflammation, including EAU and EIU (Xu et al., 2007; Toguri et al., 2015). Activation of cannabinoid receptors, specifically CB_2_R, results in decreases in immune cell migration (during both innate and adaptive immune responses), T-cell proliferation, inflammatory mediator release and alterations in local blood flow. Furthermore, there are indications that cannabinoids may have comparable and, in some cases, superior efficacy and less side-effects compared to traditional immunosuppressive therapeutics used in the clinic (Toguri et al., 2014). In addition to an anti-inflammatory role for cannabinoids in uveitis, this class of drugs, particularly cannabinoids that act at CB_1_R, may have therapeutic relevance for corneal surface damage and pain. However, at this time research on corneal ECS is still in its infancy and further investigation of the corneal ECS and the effects of cannabinoids in models of corneal disease, including CNP, must be conducted in order to better clarify cell and receptor targets and identify how alterations in the ECS affect corneal function (Yang et al., 2010, 2013; Murataeva et al., 2015).

Furthermore, while topical and regional cannabinoid therapies offer several advantages for treating ocular inflammation and pain, it should also be noted that there are challenges in formulation of these very lipophilic compounds for drug delivery and their use may result in dose-dependent ocular toxicity including hyperemia and reduction in tear production, as well as tachyphylaxis with chronic use (Green and Roth, 1982; Jay and Green, 1983). Future research should explore novel cannabinoid drug combinations that maximize efficacy and limit dose including allosteric modulators (Cairns et al., 2016b), as well as appropriate routes of local delivery, novel drug formulations and studies of both acute and chronic dosing in representative models of ocular disease.

## Author Contributions

JT wrote the majority of this manuscript. MC contributed to writing and editing this manuscript. MK contributed to writing and editing this manuscript.

## Conflict of Interest Statement

MK is a founding director and CSO of Panag Pharma Inc., a start-up company developing cannabinoid therapeutics for pain and inflammation. JT is a post-doctoral fellow for Panag Pharma Inc. The other author declares that the research was conducted in the absence of any commercial or financial relationships that could be construed as a potential conflict of interest.

## Abbreviations

2-AG: 2-arachidonoyl glycerol
BCEC: Bovine corneal epithelial cell
CB_1_R: cannabinoid 1 receptor
CB_1_R^-/-^: cannabinoid 1 receptor knockout
CB_2_R: cannabinoid 2 receptor
CNS: central nervous system
DC: dendritic cell
EAU: experimental autoimmune uveoretinitis
ECS: endocannabinoid system
EGFR: epidermal growth factor receptor
EIU: endotoxin-induced uveitis
ERK: extracellular signal-regulated kinases
HCEC: human corneal epithelial cells
IOP: intraocular pressure
TRPV1: transient receptor potential channel subtype 1
Vc/C1: subnucleus caudalis/upper cervical cord
Vi/Vc: subnucleus interpolaris/ subnucleus caudalis
